# Supplementary material for: Polymorphisms and Gene-Gene Interaction in AGER/IL6 Pathway Might Be Associated with Diabetic Ischemic Heart Disease
Source: J Pers Med. 2022 Mar 4;12(3):392. doi: 10.3390/jpm12030392 (PMC8950247; doi:10.3390/jpm12030392)
Supplement: Supplementary file 1 [file jpm-12-00392-s001.zip › jpm-1545818-supplementary-update.pdf]

Table S1 General information of the candidate SNPs and their primer sequences

Gene	Rs number	Relevant trait/ Population/ Reference	Location	Minor/Major allele	MAF	Primer name	Primer sequence
AGER	rs1035798	Ischemic stroke	intron	A/G	0.164	Forward	5'-ACGTTGGATGAAAAAGCCTTCAACCCCAGC-3'
		Swedish [1]				Reverse	5'-ACGTTGGATGCTGAAGGAGGAAAAATCCAG-3'
						UEP-SEQ	5'-GGTGGTGAAGGTTCTCAAACTCTGT-3'
	rs1800624	Ulcerative colitis	promoter	T/A	0.166	Forward	5'-ACGTTGGATGGCCAGACTGTTGTCTGCAAG-3'
		Chinese [2]				Reverse	5'-ACGTTGGATGAAAACATGAGAAACCCCAG-3'
						UEP-SEQ	5'-GCATTGCCTTCATGATGCAGGCCCAA-3'
	rs1800625	Ulcerative colitis	promoter	G/A	0.129	Forward	5'-ACGTTGGATGGCCAGACTGTTGTCTGCAAG-3'
		Chinese [2]				Reverse	5'-ACGTTGGATGAAAACATGAGAAACCCCAG-3'
						UEP-SEQ	5'-GAACAAAAATGATTTTCTTTCACGAAG-3'
	rs184003	Asthma, diabetes and its complications	intron	G/T	0.152	Forward	5'-ACGTTGGATGTTTCCCTCGTTAGCCCTCTG-3'
		Chinese [3,4]				Reverse	5'-ACGTTGGATGCACTGGATGAAGGATGTGAG-3'
						UEP-SEQ	5'-GTAGGGTGAACCATAACTA-3'
	rs2070600	Diabetic nephropathy, cardiovascular disease	Exon-missense	T/C	0.105	Forward	5'-ACGTTGGATGCCATTCCTGTTCATTGCCTG-3'
		Finnish, American [5,6]				Reverse	5'-ACGTTGGATGACAGTGTGGCTCGTGTCCTT-3'
						UEP-SEQ	5'-GCTTGCTCGTGTCCTTCCCAAC-3'
IL6R	rs2228144	Ischemic stroke	Exon-synonymous	A/G	0.096	Forward	5'-ACGTTGGATGAAGTGTCTTCTCCCTCCTCC-3'
		Korean [7]				Reverse	5'-ACGTTGGATGAACAGTGGCATTGTCTTCCG-3'
						UEP-SEQ	5'-AGGGTCAGCACGCCTCT-3'
	rs4072391	Lung cancer	3' UTR	T/C	0.398	Forward	5'-ACGTTGGATGCCACCCTCTCAAGAGAAAAC-3'

		/ miRNA binding site				
	Chinese [8]				Reverse	5'-ACGTTGGATGAACCTGGGTAACTAGGGAAG-3'
					UEP-SEQ	5'-GAGCCTGGCAAGAATG-3'
rs4129267	Cardiovascular disease American [9]	intron	T/C	0.401	Forward	5'-ACGTTGGATGGGATCTCAGATGAGGTGATG-3'
					Reverse	5'-ACGTTGGATGCAGTCTAGAGGTGGAAATGG-3'
					UEP-SEQ	5'-CGGAGTGGGGTCAATTCT-3'
rs4537545	Inflammation French [10]	intron	T/C	0.498	Forward	5'-ACGTTGGATGAGTAAGGACTAGCAAGGAGG-3'
					Reverse	5'-ACGTTGGATGTTTCCCCCTTACTGGTGATG-3'
					UEP-SEQ	5'-GGAAACCCTCCCTGA-3'
rs4845625	Dyslipidemia Japanese [11]	intron	T/C	0.14	Forward	5'-ACGTTGGATGGAAGCAGCTGTTGTCCTTAC-3'
					Reverse	5'-ACGTTGGATGTCTGTCCAAGGTGACATAGC-3'
					UEP-SEQ	5'-GAACCAGCATTCCAGTCA-3'
rs7514452	Obesity Indian [12]	3' UTR/ miRNA binding site	C/T	0.096	Forward	5'-ACGTTGGATGGGTTTCAAACCTCCCTTTCC-3'
					Reverse	5'-ACGTTGGATGGTGTGAGTGTCCAAAGAGTC-3'
					UEP-SEQ	5'-GGGCCCAAGTTCACTCTA-3'
rs7529229	Coronary heart disease Chinese [13]	intron	C/T	0.397	Forward	5'-ACGTTGGATGTTCCCTTTCACAGAGGTTTG-3'
					Reverse	5'-ACGTTGGATGCTGATGCCAGACCGAATTTG-3'
					UEP-SEQ	5'-GAACTGTGGTCGTGGTGAGTTACC-3'

MAF minor allele frequency, *SNP* single nucleotide polymorphism;

Reference:

[1] Olsson, S.; Jood, K. Genetic variation in the receptor for advanced glycation end-products (RAGE) gene and ischaemic stroke. *European Journal of Neurology* **2013**, 20, 991-3.
[2] Wang, Z.T.; Wang, L.Y.; Wang, L.; Cheng, S.; Fan, R.; Zhou, J.; Zhong, J. Association between RAGE gene polymorphisms and ulcerative colitis susceptibility: a case-control study in a Chinese Han population. *Genetics & Molecular Research Gmr* **2015**, 14, 19242.

[3] Kang, P.; Tian, C.; Jia, C. Association of RAGE gene polymorphisms with type 2 diabetes mellitus, diabetic retinopathy and diabetic nephropathy. *Gene* **2012**, 500, 1-9.
[4] Niu, H.; Niu, W.; Yu, T.; Dong, F.; Huang, K.; Duan, R.; Qumu, S.; Lu, M.; Li, Y.; Yang, T.; Wang, C. Association of RAGE gene multiple variants with the risk for COPD and asthma in northern Han Chinese. *Aging (Albany NY)* **2019**, 11, 3220-3237.
[5] Wadén, J.M.; Dahlström, E.H.; Elonen, N.; Thorn, L.M.; Wadén, J.; Sandholm, N.; Forsblom, C.; Groop, P.H.; FinnDiane Study Group. Soluble receptor for AGE in diabetic nephropathy and its progression in Finnish individuals with type 1 diabetes. *Diabetologia* **2019**, 62, 1268-1274.
[6] Maruthur, N.M.; Li, M.; Halushka, M.K.; Astor, B.C.; Pankow, J.S.; Boerwinkle, E.; Coresh, J.; Selvin, E.; Kao, W.H. Genetics of Plasma Soluble Receptor for Advanced Glycation End-Products and Cardiovascular Outcomes in a Community-based Population: Results from the Atherosclerosis Risk in Communities Study. *PLoS One* **2015**, 10, e0128452.
[7] Kim, D.H.; Yoo, S.D.; Chon, J.; Yun, D.H.; Kim, H.S.; Park, H.J.; Kim, S.K.; Chung, J.H.; Kang, J.K.; Lee, S.A. Interleukin-6 Receptor Polymorphisms Contribute to the Neurological Status of Korean Patients with Ischemic Stroke. *J Korean Med Sci* **2016**, 31, 430-4.
[8] He, F.; Yang, R.; Li, X.Y.; Ye, C.; He, B.C.; Lin, T.; Xu, X.Q.; Zheng, L.L.; Luo, W.T.; Cai, L. Single nucleotide polymorphisms of the NF-κB and STAT3 signaling pathway genes predict lung cancer prognosis in a Chinese Han population. *Cancer Genet* **2015**, 208, 310-8.
[9] Key, K.V.; Mudd-Martin, G.; Moser, D.K.; Rayens, M.K.; Morford, L.A. Inflammatory Genotype Moderates the Association Between Anxiety and Systemic Inflammation in Adults at Risk for Cardiovascular Disease. *J Cardiovasc Nurs* **2022**, 37, 64-72.
[10] Arguinano, A.A.; Naderi, E.; Ndiaye, N.C.; Stathopoulou, M.; Dadé, S.; Alizadeh, B.; Visvikis-Siest, S. IL6R haplotype rs4845625*T/rs4537545*C is a risk factor for simultaneously high CRP, LDL and ApoB levels. *Genes Immun* **2017**, 18, 163-169.
[11] Abe, S.; Tokoro, F.; Matsuoka, R.; Arai, M.; Noda, T.; Watanabe, S.; Horibe, H.; Fujimaki, T.; Oguri, M.; Kato, K.; Minatoguchi, S.; Yamada, Y. Association of genetic variants with dyslipidemia. *Mol Med Rep* 2015, 12, 5429-36.
[12] Tabassum, R.; Mahendran, Y.; Dwivedi, O.P.; Chauhan, G.; Ghosh, S.; Marwaha, R.K.; Tandon, N.; Bharadwaj, D. Common variants of IL6, LEPR, and PBEF1 are associated with obesity in Indian children. *Diabetes* **2012**, 61, 626-31.
[13] He, F.; Teng, X.; Gu, H.; Liu, H.; Zhou, Z.; Zhao, Y.; Hu, S.; Zheng, Z. Interleukin-6 receptor rs7529229 T/C polymorphism is associated with left main coronary artery disease phenotype in a Chinese population. *Int J Mol Sci* **2014**, 15, 5623-33.

Table S2 Recommendation cutoffs and proportions according to cutoffs in healthy people and ischemic heart disease patients with diabetes

		Controls [1]	T2DM+IHD [2]	*P* value
SBP	Ideal cutoffs (mmHg)	≤140	≤130	
	Ideal proportion, n (%)	509(57.8)	73(36.3)	<0.001**
DBP	Ideal cutoffs (mmHg)	≤130	≤140	
	Ideal proportion, n (%)	749(85.2)	58(28.9)	<0.001**
TC	Ideal cutoffs (mmol/L)	<5.2	<4.5	
	Ideal proportion, n (%)	471 (53.4)	81 (44.4)	0.022*
HDLC	Ideal cutoffs (mmol/L)	>1.0	>1.0 (male); >1.3(female)	
	Ideal proportion, n (%)	772 (87.5)	124 (63.3)	<0.001**
LDLC	Ideal cutoffs (mmol/L)	<3.4	<1.8	
	Ideal proportion, n (%)	596 (67.6)	24 (12.2)	<0.001**
TG	Ideal cutoffs (mmol/L)	<1.7	<1.7	
	Ideal proportion, n (%)	575 (65.2)	55 (28.1)	<0.001**

T2DM type 2 diabetes, *IHD* ischemic heart disease, *SBP* systolic blood pressure, *DBP* diastolic blood pressure, *TG* triglyceride, *TC* total cholesterol, *LDL-C* low density lipoprotein cholesterol, *HDL-C* high density lipoprotein cholesterol.

[1] Ideal cutoffs in controls (healthy people) were from "The 2016 Chinese guidelines for the management of dyslipidemia in adults".

[2] Ideal cutoffs in cases (diabetic ischemic heart disease patients) were from "The 2017 Guidelines for the prevention and treatment of type 2 diabetes in China".

**$P<0.01$, * $P<0.05$.

Table S3 Associations of gene polymorphisms with the risk of diabetic cardiovascular disease

	Genotype	Crude OR [1] (95%CI)	Crude P value	Adjusted OR¤ (95%CI)	Adjusted P value
rs1035798	GG	Ref	Ref	Ref	Ref
	AG	1.156 (0.817, 1.637)	0.413	1.240 (0.817, 1.883)	0.313
	AA	0.887 (0.360, 2.183)	0.794	0.790 (0.225, 2.449)	0.683
	additive	1.069 (0.805, 1.420)	0.645	1.096 (0.777, 1.546)	0.6
	dominant	1.124 (0.804, 1.570)	0.495	1.185 (0.791, 1.775)	0.411
	recessive	0.852 (0.348, 2.086)	0.725	0.741 (0.241, 2.281)	0.602
rs1800624	AA	Ref	Ref	Ref	Ref
	AT	1.105 (0.776, 1.572)	0.58	1.206 (0.790, 1.841)	0.385
	TT	0.987 (0.446, 2.183)	0.974	0.949 (0.365, 2.467)	0.915
	additive	1.054 (0.799, 1.389)	0.711	1.095 (0.787, 1.524)	0.589
	dominant	1.088 (0.778, 1.521)	0.622	1.168 (0.780, 1.747)	0.451
	recessive	0.961 (0.437, 2.114)	0.921	0.900 (0.349, 2.321)	0.828
rs1800625	AA	Ref	Ref	Ref	Ref
	AG	1.071 (0.746, 1.538)	0.709	1.356 (0.877, 2.097)	0.171
	GG	2.324 (0.672, 8.041)	0.183	2.373 (0.527, 10.677)	0.26
	additive	1.157 (0.835, 1.601)	0.381	1.396 (0.945, 2.062)	0.094
	dominant	1.117 (0.785, 1.589)	0.539	1.399 (0.914, 2.140)	0.122
	recessive	2.286 (0.663, 7.885)	0.191	2.204 (0.493, 9.851)	0.301
rs2228144	GG	Ref	Ref	Ref	Ref
	AG	1.301 (0.889), 1.904	0.175	1.118 (0.687, 1.818)	0.654
	AA	1.679 (0.520, 5.425)	0.386	1.534 (0.387, 6.090)	0.543
	additive	1.300 (0.935, 1.806)	0.118	1.154 (0.765, 1.742)	0.495
	dominant	1.326 (0.917, 1.917)	0.134	1.149 (0.720, 1.834)	0.559
	recessive	1.594 (0.495, 5.135)	0.435	1.504 (0.380, 5.951)	0.561
rs4072391	CC	Ref	Ref	Ref	Ref
	CT	1.149 (0.777, 1.698)	0.487	1.177 (0.728, 1.903)	0.506
	TT	0.675 (0.081, 5.644)	0.717	-	-
	additive	1.300 (0.935, 1.806)	0.118	1.048 (0.666, 1.650)	0.838
	dominant	1.129 (0.767, 1.662)	0.537	1.118 (0.693, 1.802)	0.647
	recessive	0.658 (0.079, 5.493)	0.658	-	-
rs4129267	CC	Ref	Ref	Ref	Ref
	CT	1.327 (0.937, 1.877)	0.111	1.323 (0.865, 2.023)	0.197
	TT	1.430 (0.898, 2.279)	0.132	1.575 (0.891, 2.783)	0.118
	additive	1.217 (0.973, 1.521)	0.085	1.266 (0.961, 1.667)	0.094
	dominant	1.351 (0.970, 1.880)	0.075	1.378 (0.919, 2.067)	0.121
	recessive	1.208 (0.799, 1.825)	0.371	1.329 (0.803, 2.199)	0.268
rs4537545	CC	Ref	Ref	Ref	Ref
	CT	1.395 (0.984, 1.978)	0.062	1.485 (0.966, 2.283)	0.071
	TT	1.386 (0.866, 2.220)	0.174	1.585 (0.887, 2.833)	0.12
	additive	1.217 (0.973, 1.521)	0.085	1.292 (0.980, 1.705)	0.07

	dominant	1.393 (0.998, 1.944)	0.051	1.507 (0.998, 2.276)	0.051
	recessive	1.132 (0.747, 1.716)	0.558	1.239 (0.745, 2.060)	0.409
rs7514452	TT	Ref	Ref	Ref	Ref
	CT	1.113 (0.751, 1.650)	0.594	1.136 (0.700, 1.846)	0.605
	CC	0.671 (0.080, 5.610)	0.712	-	-
	additive	1.069 (0.739, 1.544)	0.724	1.015 (0.642, 1.605)	0.95
	dominant	1.095 (0.742, 1.616)	0.647	1.079 (0.666, 1.748)	0.757
	recessive	0.658 (0.079, 5.493)	0.699	-	-
rs7529229	TT	Ref	Ref	Ref	Ref
	CT	1.289 (0.912, 1.822)	0.151	1.301 (0.851, 1.989)	0.225
	CC	1.403 (0.881, 2.234)	0.153	1.557 (0.881, 2.752)	0.127
	additive	1.069 (0.739, 1.544)	0.724	1.256 (0.953, 1.656)	0.105
	dominant	1.315 (0.946, 1.829)	0.103	1.357 (0.905, 2.034)	0.14
	recessive	1.206 (0.798, 1.822)	0.375	1.327 (0.802, 2.195)	0.271

[1] No variables were adjusted in logistic regression model ¤ Dyslipidemia, hypertension, smoking, and drinking were adjusted in the logistic regression model. Adjusted p-values shown in the table are adjusted only by covariates.

Table S4 Sensitivity analysis for the association between AGER and IL6R gene polymorphisms and diabetic ischemic heart disease

	rs4845625		rs184003		Interaction	
	OR (95%CI)	*P*	OR (95%CI)	*P*	OR (95%CI)	*P*
Crude model	0.707 (0.563, 0.888)	**0.003**	1.491 (1.125, 1.976)	**0.005**	1.381 (1.126, 1.693)	**0.002**
Model 1	0.653 (0.498, 0.858)	**0.002**	1.679 (1.200, 2.349)	**0.002**	1.166 (0.893, 1.521)	0.259
Model 2	0.729 (0.579, 0.918)	**0.007**	1.425 (1.070, 1.899)	**0.015**	1.360 (1.104, 1.676)	**0.004**
Model 3	0.713 (0.566, 0.897)	**0.004**	1.424 (1.069, 1.896)	**0.016**	1.124 (0.897, 1.408)	**0.311**
Model 4	0.714 (0.550, 0.927)	**0.011**	1.343 (0.963, 1.873)	0.082	1.291 (1.007, 1.656)	**0.044**
Model 5	0.708 (0.544, 0.920)	**0.010**	1.309 (0.935, 1.834)	0.117	1.294 (1.005, 1.667)	**0.045**

Model 1, adjust for fasting plasma glucose; model 2, adjust for hyperlipidaemia; model 3, adjust for hypertension; model 4, adjust for smoking behavior; model5, adjust for alcohol drinking behavior.

Table S5 Subgroup analysis for the association between candidate SNPs and diabetic IHD

Stratification factors		Subgroup 1		Subgroup 2	
		OR (95%CI)	P	OR (95%CI)	P
FPG [1]	rs4845625	1.170 (0.182, 7.505)	0.869	0.496 (0.288, 0.856)	**0.012**
	rs184003	1.796 (0.371, 8.694)	0.496	1.095 (0.508, 2.360)	0.817
	Gene-gene interaction	2.170 (0.654, 7.197)	0.205	0.713 (0.387, 1.313)	0.278
ICVD scores [2]	rs4845625	0.389 (0.197, 0.768)	**0.007**	0.776 (0.406, 1.483)	0.443
	rs184003	1.904 (0.839, 4.321)	0.124	0.570 (0.225, 1.445)	0.236
	Gene-gene interaction	1.071 (0.562, 2.041)	0.834	0.629 (0.300, 1.316)	0.218

[1] When stratified by FPG, subgroup 1: FPG ≤ 6.1 mmol/L, subgroup 2: FPG > 6.1 mmol/L. All models were adjusted for hyperlipidaemia, hypertension, smoking and alcohol drinking behavior.

[2] When stratified by ICVD scores, subgroups were defined according to the median of ICVD risk scores (9 points), subgroup 1: ICVD risk scores ≤ 8, subgroup 2: ICVD risk scores > 9.

Table S6 The association between candidate SNPs and FPG or ICVD risk scores

		Crude		Adjust model [3]	
		OR (95%CI)	P	OR (95%CI)	P
FPG [1]	rs4845625	0.892 (0.666, 1.195)	0.444	0.892 (0.652, 1.219)	0.472
	rs184003	0.953 (0.640, 1.417)	0.810	1.057 (0.681, 1.641)	0.805
Score [2]	rs4845625	0.879 (0.646, 1.196)	0.411	0.895 (0.634, 1.263)	0.527
	rs184003	1.135 (0.741, 1.738)	0.560	1.252 (0.769, 2.037)	0.366

[1] FPG >6.1 mmol/L was assigned to 1, and FPG ≤ 6.1 mmol/L was assigned to 0.

[2] ICVD risk socre > 9 was assigned to 1, and ICVD risk socre ≤ 9 was assigned to 0.

[3] Hyperlipidaemia, hypertension, smoking and alcohol drinking behavior were adjusted.

Table S7 The association of traits and haplotypes constituted by rs184003-rs1035798-rs2070600-rs1800624

Haplotypes	C-A-C-T		C-G-T-A		A-G-C-A		C-G-C-A	
	β	*P*	*β*	*P*	*β*	*P*	*β*	*P*
TG (mmol/L)	-0.047	0.481	-0.039	0.532	0.104	0.140	-0.005	0.919
TC (mmol/L)	-0.003	0.965	-0.026	0.663	-0.033	0.627	0.038	0.443
HDLC (mmol/L)	0.039	0.089	0.005	0.809	-0.026	0.283	-0.011	0.530
LDLC (mmol/L)	-0.016	0.743	-0.013	0.774	-0.016	0.765	0.026	0.500
SBP (mmHg)	1.082	0.272	0.338	0.709	-1.891	0.067	0.095	0.898
DBP (mmHg)	-0.161	0.789	0.148	0.788	-0.333	0.596	0.221	0.626
FPG (mmol/L)	0.122	0.663	-0.754	**0.002**	-0.130	0.618	0.512	**0.011**
AGEs (mmol/L)	-1.253	0.441	0.369	0.803	2.911	0.081	-0.971	0.423
IL6 (mmol/L)	-6.808	0.075	3.867	0.267	2.527	0.522	0.386	0.893

BP systolic blood pressure, *DBP* diastolic blood pressure, *FPG* fasting plasma glucose, *TG* triglyceride, *TC* total cholesterol, *LDL-C* low density lipoprotein cholesterol, *HDL-C* high density lipoprotein cholesterol, *AGEs* advanced glycation end products, *IL-6* interleukin 6.

Table S8 The association of traits and haplotypes constituted by rs7529229-rs4845625-rs4129267-rs7514452-rs4072391

Haplotypes	T-T-C-C-T		C-C-T-T-C		T-C-C-T-C		T-T-C-T-C	
	β	*P*	*β*	*P*	*β*	*P*	*β*	*P*
TG (mmol/L)	-0.063	0.483	0.075	0.155	0.064	0.448	-0.085	0.114
TC (mmol/L)	-0.182	**0.034**	0.055	0.281	-0.124	0.127	0.054	0.294
HDLC (mmol/L)	-0.042	0.167	0.007	0.708	0.010	0.734	0.000	0.992
LDLC (mmol/L)	-0.102	0.128	0.024	0.548	-0.115	0.067	0.057	0.150
SBP (mmHg)	1.857	0.156	-0.149	0.847	-0.640	0.603	-0.254	0.746
DBP (mmHg)	-0.548	0.492	0.469	0.318	-0.590	0.431	-0.054	0.910
FPG (mmol/L)	-0.239	0.492	-0.072	0.730	0.027	0.907	0.278	0.357
AGEs (mmol/L)	-0.079	0.970	-1.459	0.236	0.608	0.643	2.424	0.211
IL6 (mmol/L)	-1.695	0.730	0.594	0.838	0.234	0.940	-0.572	0.901

SBP systolic blood pressure, *DBP* diastolic blood pressure, *FPG* fasting plasma glucose, *TG* triglyceride, *TC* total cholesterol, *LDL-C* low density lipoprotein cholesterol, *HDL-C* high density lipoprotein cholesterol, *AGEs* advanced glycation end products, *IL-6* interleukin 6.

Table S9 The function of *AGER* and *IL6R* polymorphisms

Rs number	Location	Functional Allele	Function description
rs1035798	intron	T allele	Nominally decrease AGEs level [1]
rs1800624	promoter	A allele	Higher expression of sRAGE, AGER [1]
rs1800625	promoter	C allele	Higher expression of sRAGE, esRAGE and AGEs [1]
rs184003	intron	T allele	Higher expression of sRAGE [1]
rs2070600	Exon-missense	A allele	Rs2070600 SNP is located in the ligand-binding V domain of AGER, induces an increase in RAGE affinity for ligand AGEs. Lower level of sRAGE and AGEs [1]
rs2228144	Exon-synonymous	-	Absolute or strong linkage disequilibrium to rs2228145 which is eQTL for sIL-6R [2]
rs4072391	3' UTR/ miRNA binding site	T allele	Associated with lower plasma IL-6 concentration [3]
rs4129267	intron	T allele	Associated with higher plasma IL-6 concentration [4]
rs4537545	intron	T allele	Accounts for 20% of the variance in sIL-6R, associated with higher circulating IL-6 levels [3,5]
rs4845625	intron	T allele	Explain 55% IL-6 soluble receptor (LOD=8.8), associated with higher hsCRP and Complement C3 [6-8]
rs7514452	3' UTR/miRNA binding site	-	Downstream transcript variant, construct haplotypes associated with sIL-6R [9]
rs7529229	intron	C allele	Increased circulating interleukin-6 concentration [10]

SNPs single nucleotide polymorphisms, *AGEs* Advanced Glycation End Products, *sRAGE* soluble receptor of Advanced Glycation End Product, *AGER* Advanced Glycation End Product Receptor, *esRAGE* endogenous secretory receptor for advanced glycation end products, *eQTL* expression Quantitative Trait Loci, *sIL-6R* soluble Interleukin-6 Receptor, *IL-6* Interleukin 6, *hsCRP* hypersensitive C Reactive Protein.

Reference:

[1] Li, T.; Qin, W.; Liu, Y.; Li, S.; Qin, X.; Liu, Z. Effect of RAGE gene polymorphisms and circulating sRAGE levels on susceptibility to gastric cancer: a case-control study. *Cancer Cell Int* **2017**, 17, 19.

[2] Van Dongen, J.; Jansen, R.; Smit, D.; Hottenga, J.J.; Mbarek, H.; Willemsen, G.; Kluft, C.; AAGC Collaborators, Penninx, B.W.; Ferreira, M.A.; Boomsma, D.I.; de Geus, E.J. The contribution of the functional IL6R polymorphism rs2228145, eQTLs and other genome-wide SNPs to the heritability of plasma sIL-6R levels. *Behav Genet* **2014**,44, 368-82.

[3] Walston, J.D.; Matteini, A.M.; Nievergelt, C.; Lange, L.A.; Fallin, D.M.; Barzilai, N.; Ziv, E.; Pawlikowska, L.; Kwok, P.; Cummings, S.R.; Kooperberg, C.; LaCroix, A.; Tracy, R.P.; Atzmon, G.; Lange, E.M.; Reiner, A.P. Inflammation and stress-related candidate genes, plasma interleukin-6 levels, and longevity in older adults. *Exp Gerontol* **2009**, 44, 350-5.

[4] Naitza, S.; Porcu, E.; Steri, M.; Taub, D.D.; Mulas, A.; Xiao, X.; Strait, J.; Dei, M.; Lai, S.; Busonero, F.; Maschio, A.; Usala, G.; Zoledziewska, M.; Sidore, C.; Zara, I.; Pitzalis, M.; Loi, A.; Virdis, F.; Piras, R.; Deidda, F.; Whalen, M.B.; Crisponi, L.; Concas, A.; Podda, C.; Uzzau, S.; Scheet, P.; Longo, D.L.; Lakatta, E.; Abecasis, G.R.; Cao, A.; Schlessinger, D.; Uda, M.; Sanna, S.; Cucca, F. A genome-wide association scan on the levels of markers of inflammation in Sardinians reveals associations that underpin its complex regulation. *PLoS Genet* **2012**, 8, e1002480.

[5] Rafiq, S.; Frayling, T.M.; Murray, A.; Hurst, A.; Stevens, K.; Weedon, M.N.; Henley, W.; Ferrucci, L.; Bandinelli, S.; Corsi, A.M.; Guralnik, J.M.; Melzer, D. A common variant of the interleukin 6 receptor (IL-6r) gene increases IL-6r and IL-6 levels, without other inflammatory effects. *Genes Immun* **2007**, 8, 552-9.

[6] Reich, D.; Patterson, N.; Ramesh, V.; De Jager, P.L.; McDonald, G.J.; Tandon, A.; et al. Myocardial Infarction Genetics and CARDIoGRAM Exome Consortia Investigators. Systematic Evaluation of Pleiotropy Identifies 6 Further Loci Associated With Coronary Artery Disease. J Am Coll Cardiol. 2017 Feb 21;69(7):823-836.

[8] Christiansen, M.K.; Larsen, S.B.; Nyegaard, M.; Neergaard-Petersen, S.; Ajjan, R.; Würtz, M.; Grove, E.L.; Hvas, A.M.; Jensen, H.K.; Kristensen, S.D. Coronary artery disease-associated genetic variants and biomarkers of inflammation. *PLoS One* **2017**,12, e0180365.

[9] Gigante, B.; Strawbridge, R.J.; Velasquez, I.M.; Golabkesh, Z.; Silveira, A.; Goel, A.; Baldassarre, D.; Veglia, F.; Tremoli, E.; Clarke, R.; Watkins, H.; Hamsten, A.; Humphries, S.E.; de Faire, U. Analysis of the role of interleukin 6 receptor haplotypes in the regulation of circulating levels of inflammatory biomarkers and risk of coronary heart disease. *PLoS One* **2015**, 10, e0119980.

[10] Webb, T.R.; Erdmann, J.; Stirrups, K.E.; Stitziel, N.O.; Masca, N.G.; Jansen, H.; et al. Myocardial Infarction Genetics and CARDIoGRAM Exome Consortia Investigators. Systematic Evaluation of Pleiotropy Identifies 6 Further Loci Associated With Coronary Artery Disease. *J Am Coll Cardiol* **2017**, 69, 823-836.
